# Attitudes of the general public toward community pharmacy services in Saudi Arabia: A cross-sectional study

**DOI:** 10.3389/fpubh.2023.1092215

**Published:** 2023-02-24

**Authors:** Mona Almanasef

**Affiliations:** Department of Clinical Pharmacy, College of Pharmacy, King Khalid University, Abha, Saudi Arabia

**Keywords:** community pharmacy, public perception, public attitudes, Saudi Arabia, services

## Abstract

**Aim:**

This research was conducted to evaluate the attitudes of the general public toward community pharmacy services in Saudi Arabia.

**Methods:**

The current study followed a cross-sectional design using an online anonymous self-administered questionnaire. The study was conducted in Saudi Arabia between February and April 2022. The study participants were selected and recruited using a convenience sampling strategy. The questionnaire was adapted from previous work and involved four sections: demographic information, use of community pharmacy, perception and willingness to use community pharmacist services, and barriers to approaching a community pharmacist for advice.

**Results and conclusions:**

A total of 449 participants agreed to take part in this study and completed the questionnaire. Just above half (55%) of the participants indicated that they had visited a community pharmacy at least once a month over the last 12 months. The most frequently reported reason for visiting community pharmacies was to buy over-the-counter (OTC) medications. The majority (*n* = 318) of the participants were satisfied or highly satisfied with community pharmacy services in Saudi Arabia. A willingness to use community pharmacy services was expressed mostly for the essential community pharmacist roles, involving selecting over-the-counter products, management of minor ailments, selecting non-pharmaceuticals and receiving counseling on using medications. However, an unwillingness was noted to approach a community pharmacist for counseling on alcohol dependence and drug misuse. Lack of privacy in the community pharmacy was found to be the most frequently reported barrier that would hinder individuals from approaching community pharmacists to get help or advice. Policymakers in Saudi Arabia could considerably advance the community pharmacy practice and improve the public utilization of various community pharmacist services beyond their limited essential roles such as dispensing and counseling.

## Introduction

The community pharmacy is recognized as the most accessible healthcare sector and is utilized by a large proportion of the population (1, 2). Community pharmacists are approachable, without the need to schedule an appointment or get a referral (3). The services provided in community pharmacies are either product-oriented, such as dispensing medicines, or patient-oriented, such as providing pharmaceutical care (4, 5).

The community pharmacy sector in Saudi Arabia has grown rapidly and is now the largest employment sector in the country (6). Thus, the government has recently paid more attention to the advancement of this sector (7). Saudi Vision 2030 has emphasized public-private partnerships to ensure effective delivery of primary healthcare (3, 7–9). In addition, the Vision aims to shift the focus of healthcare from therapeutic care to primary and preventative care (8, 9).

The community pharmacy sector in Saudi Arabia is regulated by the Ministry of Health (MoH) (7). Pharmacies are usually run by one to two pharmacists, or an assistant and one pharmacist (10). The typical working hours are between 8 and 12, 6 days per week (10). However, some community pharmacies operate 24 h, 7 days per week (10).

The involvement of community pharmacists in improving the health awareness of the public and optimizing the use of medicines could contribute to reducing morbidity and mortality from chronic diseases, such as diabetes mellitus and ischemic heart disease (3). In Saudi Arabia, as suggested by previous reports, consumers approach their local community pharmacy for various reasons, including buying over-the-counter (OTC) or cosmetic products, asking the pharmacist's advice on disease-related issues, and getting prescription medications for the management of chronic illnesses (3).

According to the pharmaceutical care framework, the professional relationship between patient and pharmacist is primarily dependent on five elements, i.e., caring, trust, communication, cooperation, and mutual decision-making (1, 5). The patient's understanding the community pharmacist's role is paramount for the successful implementation of the pharmaceutical care framework (5).

The community pharmacy practice in Saudi Arabia has witnessed a tremendous expansion in services, with a focus on patient-centered care due to the recent transformation of the healthcare system (3, 7). In addition to the traditional services provided, a number of pharmacist-led services are now being offered in large chain community pharmacies, and this involves vaccinations, diabetes management programmes, weight management programmes, and vital signs and biomarker measurements (9).

Evaluation of patient satisfaction with the services provided is important for various reasons (11). Higher levels of patient satisfaction reflect the superior quality of the healthcare services provided (11). What is more, patient perception is key for improving the quality of the existing services, assessing the need for additional services and improving patient-pharmacist communication and expectations (4).

To the best of our knowledge, there is a lack of studies that evaluate general public attitudes toward community pharmacy services in Saudi Arabia. Hence, this cross-sectional study was conducted to investigate the attitudes of the general public toward community pharmacy services in Saudi Arabia, given the recent expansion in the services provided.

## Methods

### Study design and setting

The study followed a cross-sectional design using an anonymous self-administered online questionnaire. The study took place in Saudi Arabia between February and April 2022. The study participants involved male and female residents of Saudi Arabia who are 18 years of age or older.

### Population and sampling

This research has employed a multi-phase sampling technique. First, a cluster sampling method was used where five regions in Saudi Arabia i.e., Eastern Province, Riyadh, Asir, Madinah, and Northern Borders were selected for data collection as each belong to a different part of the country. Potential participants who visited the community pharmacy were invited to take part in the study and fill out the self-administered questionnaire through five community pharmacists working in the different above-mentioned regions. Second, in attempts to increase the response rate a convenience sampling method was used which involved advertising the questionnaire link through social media platforms, including WhatsApp and Telegram groups in Saudi Arabia.

The minimum recommended sample size was estimated to be 385 participants based on a population size of 36,000,000 persons (10), with a 5% margin of error, a 95% confidence level and a 50% response distribution.

### Data collection form

The questionnaire was adapted from previous research (1, 5, 9) and it involved a total of four sections. Section One collected sociodemographic information, i.e., age group, gender, nationality, marital status and educational level. Section Two involved questions related to the use of the community pharmacy and it assessed participants' satisfaction with the community pharmacy services in Saudi Arabia. Section Three evaluated participants' willingness to use different pre-determined community pharmacy services. Section Four gathered data on barriers that would prevent patients from approaching a community pharmacist for help or advice.

The original version of the questionnaire was prepared in the English language, and then translated into Arabic. In order to ensure the validity of the translation, a back translation technique was undertaken by the study investigator and two translators who have excellent proficiency in both languages.

In order to ensure the clarity of the questions, the questionnaire was pilot-tested with seven participants who met the inclusion criteria of the study. Modifications were made on the questionnaire based on the pilot test feedback. The data from the pilot study were excluded from the final results. The final questionnaire was distributed in the Arabic language, which is the official language of Saudi Arabia.

The questionnaire was created using the Google forms platform. An introductory statement inviting eligible subjects to participate in the study, and highlighting the aim of the study and the inclusion criteria, was added to the survey link. The cover page of the questionnaire displayed a participant information sheet with detailed information about the conduct of the study.

### Statistical analysis

Data analysis was performed using the Statistical Package for Social Sciences (SPSS) version 27.0 for Mac. Descriptive statistics—i.e., frequencies and percentages—were used to present the study findings. Participants' willingness to use predetermined community pharmacist services was evaluated using a Likert scale ranging from 1 (definitely unwilling) to 5 (definitely willing). Scale distribution was presented in percentages, as well as skew, mean and standard deviation (SD). A positive value of skewness indicates skewness toward 1 (definitely unwilling). However, negative value indicates a skewness toward 5 (definitely willing). Chi square test of independence was used to examine the association between the willingness to use community pharmacy services and selected demographic variables. Pearson's correlations test was used to evaluate the relationship between barriers and willingness to use different community pharmacy services. The level of statistical significance was set at an alpha level equal to 0.05 for all analyses.

### Ethical considerations

Ethical approval was granted by the King Khalid University Research Ethics Committee, approval reference (HAPO-06-B-001). Participation in the study was voluntary and potential participants had the right to decline the invitation to participate without any penalty.

## Results

A total of 449 participants agreed to take part in this study and completed the questionnaire. Just below two thirds (64.4%) of them were females, and slightly less than half (43.7%) of them were between 18 and 30 years of age. The majority of the participants (95.5%) were Saudi nationals. Slightly less than two-thirds of the participants were married (58.1%) and held a bachelor's degree (61.5%) at the time of collecting the data (Table 1).

**Table 1:** Demographic information of the participants (*n* = 449).

**Characteristics**	***n* (%)**
**Gender**	
Male	160 (35.6)
Female	289 (64.4)
**Age group**	
18–30 years	196 (43.7)
31–45 years	143 (31.8)
46–60 years	90 (20)
61 years or older	20 (4.5)
**Nationality**	
Saudi	429 (95.5)
Non-Saudi	20 (4.5)
**Marital status**	
Married	261 (58.1)
Unmarried	188 (41.9)
**Educational level**	
High school or less	73 (16.3)
Diploma	57 (12.7)
Bachelor	276 (61.5)
Postgraduate	43 (9.6)

Just above half (55%) of the participants indicated that they had visited a community pharmacy at least once a month in the last 12 months (Table 2). The most commonly reported reason for visiting the community pharmacy was to get OTC medications (90.6%), followed by getting non-pharmaceutical products (87.3%) and prescription medications (73.7%). Location was the top rated (94%) factor influencing the choice of any particular community pharmacy. However, the lowest-rated factors were attractive pharmacy appearance (46.1%) and acquaintance with pharmacist and/or staff (31.6%). All other listed factors were reported by at least two thirds of the study participants (Table 2).

**Table 2:** Participants' use of community pharmacy services in Saudi Arabia.

**Characteristics**	***n* (%)**
**Frequency of visiting community pharmacy**	
At least once a week	78 (17.4)
At least once a month	247 (55)
At least once every 3 months	78 (17.4)
At least twice yearly	25 (5.6)
At least once yearly	21 (4.7)
**Reasons for visiting community pharmacy**	
To ask for advice	114 (25.4)
To get OTC medications	407 (90.6)
To get prescription medications	331 (73.7)
To get general health information	79 (17.6)
To get home diagnostic devices	147 (32.7)
To get non-pharmaceutical products	392 (87.3)
**Factors influencing the choice of any particular community pharmacy**	
Location	422 (94)
Good range of products and services available	369 (82.2)
Quick services	358 (79.7)
Convenient working hours	354 (78.8)
Good and competitive prices	321 (71.5)
The pharmacist's knowledge and their ability to answer any drug or disease-related question	317 (70.6)
Privacy and confidentiality	305 (67.9)
Friendliness of the pharmacy staff	304 (67.7)
Attractive pharmacy appearance	207 (46.1)
Acquaintance with pharmacist and/or staff	142 (31.6)

**Figure 1:**
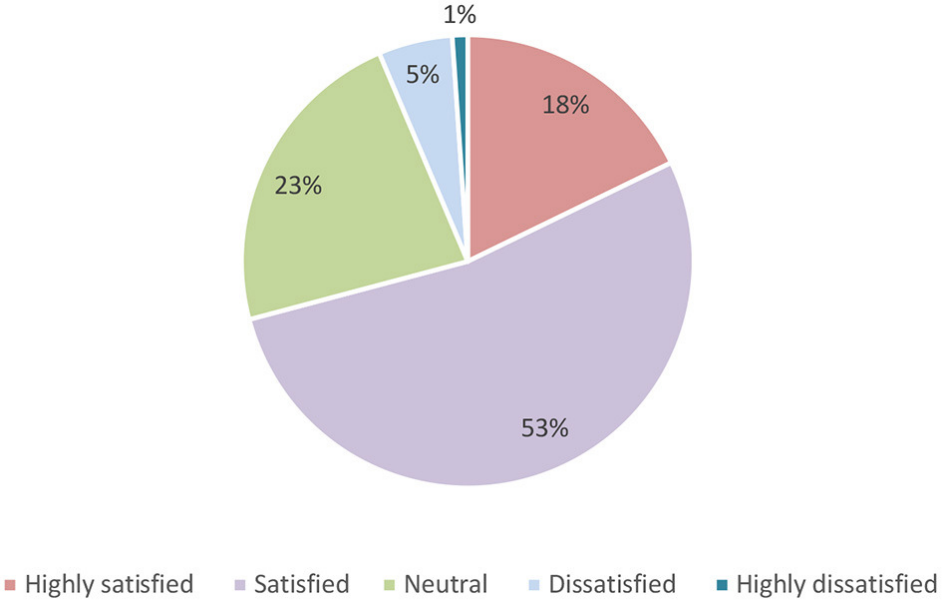
Participants' satisfaction with community pharmacy services in Saudi Arabia.

When asked about their overall satisfaction with community pharmacy services in Saudi Arabia, just above half (53%) of the participants indicated that they were satisfied, and 17.8% were highly satisfied (Figure 1).

The distribution of participants' willingness to use a predetermined community pharmacy services is presented in Table 3, where responses ranged on the scale from 1 (definitely unwilling) to 5 (definitely willing). Distribution of four community pharmacy services—i.e., selecting OTC products, management of minor ailments, selecting non-pharmaceuticals and counseling on using medications were found to be moderately skewed (between −0.1 and −0.5) toward 5 (definitely willing). However, the distribution for counseling on the alcohol dependence and drug misuse service was moderately skewed (between +0.5 and +1) toward 1 (definitely unwilling). The distribution of participants' willingness to use the following pharmacy services: medication therapy management, dental health, screening for diseases, smoking cessation, disease counseling, healthy eating, physical activity promotion, and vaccination and immunization, were relatively skewed toward 5 (definitely willing), as the skewness value was more than −0.5 but <0. However, the findings showed relative skewness toward 1 (definitely unwilling) for sexual health and weight management, as the skewness value was more than 0, but <+0.5).

**Table 3:** Distribution of participants' willingness to use community pharmacy services (*n* = 449).

**Service**	**Distribution of responses (%)**					**Skew**	**Mean**	**SD**
	**1 Definitely unwilling**	**2 Probably unwilling**	**3 Neutral**	**4 Probably willing**	**5 Definitely willing**			
Selecting over-the-counter products	7.3	8.2	17.8	26.5	40.1	−0.87	3.84	1.24
Management of minor ailments	10	6.5	18	27.6	37.9	−0.87	3.77	1.29
Selecting non-pharmaceuticals	11.4	6.5	20.5	24.7	37	−0.76	3.69	1.33
Counseling on using medications	12.2	8	22.3	26.9	30.5	−0.62	3.55	1.33
Medication therapy management	13.4	9.8	27.4	22.9	26.5	−0.41	3.39	1.33
Dental health	14	12.2	24.3	25.8	23.6	−0.37	3.33	1.34
Screening for diseases	17.4	11.6	25.2	23.2	22.7	−0.28	3.22	1.38
Smoking cessation	14	16.7	27.2	23.6	18.5	−0.17	3.16	1.29
Disease counseling	18	12.9	29.4	17.4	22.3	−0.14	3.13	1.38
Healthy eating	16.3	14	30.3	23.6	15.8	−0.175	3.09	1.29
Physical activity promotion	16.3	14.9	29.4	25.2	14.3	−0.176	3.06	1.27
Vaccination and immunization	22	12.5	27.4	17.6	20.5	−0.07	3.02	1.42
Sexual health	22.5	14.9	30.5	20	12	0.012	2.84	1.31
Weight management	20.5	21.8	29	19.4	9.4	0.128	2.75	1.24
Counseling on alcohol dependence and drug misuse	44.1	11.6	28.3	8	8	0.63	2.24	1.31

A significantly higher level of participants' willingness to approach a community pharmacist for selecting over-the-counter products, management of minor ailments, selecting non-pharmaceuticals, counseling on using medications, medication therapy management, and vaccination and immunization was observed among those who are in the younger age group when compared to those who are older than 45 years of age, *P* < 0.05 (Appendix 1).

Lack of privacy in the pharmacy was the top rated (59.2%) barrier preventing patients from approaching community pharmacists for help or advice (Table 4). This was followed by busyness of the pharmacist (49.2%), lack of awareness of the ability of the pharmacist to answer drug and disease-related questions (41.4%), and rudeness of the pharmacist (38.3%). The lowest-rated barriers were lack of trust in the pharmacist (28.5%) and fear of asking the pharmacist (26.9%).

**Table 4:** Barriers that prevent patients from approaching community pharmacists for help or advice.

**Barrier**	***n* (%)**
Lack of privacy in the pharmacy	266 (59.2)
Busyness of the pharmacist	221 (49.2)
Lack of awareness of the ability of the pharmacist to answer drug and disease-related questions	186 (41.4)
Rudeness of the pharmacist	172 (38.3)
Lack of trust in the pharmacist	128 (28.5)
Fear of asking the pharmacist	121 (26.9)

The findings showed a statistically significant positive correlation between lack of trust in the pharmacist and willingness to use most of the community pharmacy services, *P* < 0.05 (Appendix 2). This indicates that people who did not trust the community pharmacist were less likely to use community pharmacy services. Interestingly, a significant positive correlation was found between lack of privacy in the pharmacy and willingness to seek counseling on alcohol dependence and drug misuse; and between lack of awareness of the pharmacist ability and willingness to seek advice on management of minor ailments, *P* < 0.05.

## Discussion

This research was conducted to evaluate the attitudes of the general public toward community pharmacy services in Saudi Arabia. The findings from the current study showed high overall satisfaction levels with community pharmacy services in Saudi Arabia. Similar research was conducted in Qatar and Almadina city, Saudi Arabia, and reported a positive attitude toward community pharmacists and pharmacy services (5, 12). In Saudi Arabia, the community pharmacy sector has witnessed tremendous development, which has shifted the focus from product to patient-centered care (3, 4, 9, 13). This was evident in the provision of pharmacist-led services in large chain community pharmacies, such as COVID-19 and influenza vaccines, vital signs and biomarker measurements, as well as weight and diabetes management programmes (9). In addition, an e-prescribing system (Wasfaty) was launched in 2018 with an attempt to involve the private community pharmacy sector in the provision of medications (10, 14).

Consistent with previous studies, the majority of the respondents visited the community pharmacy at least once a month (5, 15–18). It is interesting to note that the top-listed reason for visiting a community pharmacy was to obtain OTC medications (90.6%). This was similar to what was reported in a research study conducted in Qatar (93%). However, the percentage was lower in other countries, i.e., Jordan (50.3%), Malta (23.3%), and the United Kingdom (11.3%) (15–17). In Saudi Arabia, there is uncontrolled access to medications, as some prescription-only medications can still be purchased from the community pharmacy without prescription (7, 19). This could explain the reason why this percentage was found to be high in the current research. Other reasons for visiting a community pharmacy found in the current research involved getting non-pharmaceutical products and prescription medications. The findings from the present study seem to be consistent with other research, which found that pharmacy location was the main factor influencing the choice of any particular community pharmacy (5, 12, 15–18).

Another important finding was that a willingness to use community pharmacy services was expressed mostly for the essential community pharmacist roles, involving selecting OTC products, management of minor ailments, selecting non-pharmaceuticals and counseling on using medications. However, an unwillingness was noted to approach a community pharmacist for counseling on alcohol dependence and drug misuse. This accords with our earlier observations, which showed that community pharmacists in Saudi Arabia had minimal involvement in the provision of counseling on alcohol dependence and drug misuse (9). This could be explained by the fact that the use of alcohol and drugs in Saudi Arabia is illegal and not permissible under Islamic law (9, 20, 21). Therefore, the fear of disclosure, and the stigma attached to alcohol and drug use, make it difficult to approach the community pharmacist for advice (9). The data from the current research support this claim as a positive correlation was observed between lack of privacy in the community pharmacy and willingness to seek counseling on alcohol dependence and drug misuse.

Interestingly, older people were found less willing than their younger counterparts to approach a community pharmacist for selecting over-the-counter products, management of minor ailments, selecting non-pharmaceuticals, counseling on using medications, medication therapy management, and vaccination and immunization. A possible explanation for this finding may be that older people are not accustomed to receiving these services from a community pharmacist.

The results of this study show that lack of privacy was the top-rated barrier preventing patients from approaching a community pharmacist for advice. What is surprising is that lack of trust of the pharmacist was one of lowest-rated barriers preventing patients from seeking advice from a community pharmacist. However, a positive correlation was found between lack of trust in the pharmacist and willingness to use community pharmacy services. In addition, willingness to seek advice on management of minor ailments was found to be positively correlated to the awareness of the ability of the pharmacist to answer drug and disease-related questions.

The healthcare system in Saudi Arabia is moving away from the traditional curative physician-governed system, to a more preventative healthcare system (8, 9). Community pharmacists are expected to have adequate therapeutic judgment and expertise, as well as a willingness to address patients' needs and concerns (5). Directing patients to use a community pharmacy for managing minor ailments would free up physicians' time to focus on treating serious health issues (5). Policymakers in Saudi Arabia could make considerable advances in community pharmacy practice and improve the public utilization of various community pharmacist services beyond the limited essential roles, such as dispensing and counseling. This could involve setting out a clear vision for the services provided at the community pharmacy level, supported by establishing relevant frameworks, in order to fully utilize the potential and expertise of pharmacists (22). Equally importantly, policymakers should facilitate the integration between primary and community healthcare services, in order to ensure smooth and effective implementation of community pharmacy services.

The findings in the current study are subject to a number of limitations. First, the male to female ratio of the respondents (1: 1.8) is not representative of the gender distribution of the population (1.3:1), thus male respondents were under-represented in the current sample. Besides, the majority of the study participants were young adults and middle-aged with higher education. Thus, the results might not be applicable to the entire population, since the elderly in the current research represent only 4.5% of the study sample.

## Conclusion

The current study demonstrated overall high satisfaction with community pharmacy services in Saudi Arabia. Lack of privacy was the top-rated barrier preventing patients from approaching a community pharmacist for advice. Willingness to use community pharmacy services was expressed mostly for the essential community pharmacist roles, and therefore policymakers in Saudi Arabia could enhance the public utilization of various community pharmacist services beyond the limited essential roles, such as dispensing and counseling. This could be achieved by establishing relevant frameworks, as well as facilitating the integration of primary and community healthcare services, to ensure smooth and effective implementation of community pharmacy services.

## Data availability statement

The original contributions presented in the study are included in the article/Supplementary material, further inquiries can be directed to the corresponding author.

## Ethics statement

Ethical approval was granted by the King Khalid University Research Ethics Committee, approval reference (HAPO-06-B-001). The patients/participants provided their written informed consent to participate in this study. Written informed consent was obtained from the individual(s) for the publication of any potentially identifiable images or data included in this article.

## Author contributions

MA has contributed to the design of the study, data collection, data analysis, and manuscript write up. The author confirms being the sole contributor of this work and has approved it for publication.

## References

[1] Awad AI Al-Rasheedi A Lemay J. Public perceptions, expectations, and views of community pharmacy practice in Kuwait. Med Princ Pract. (2017) 26:438–46. 10.1159/000481662 28934755 PMC5757534

[2] Alfadl AA Alrasheedy AA Alhassun MS. Evaluation of medication counseling practice at community pharmacies in Qassim region, Saudi Arabia. Saudi Pharm J. (2018) 26:258–62. 10.1016/j.jsps.2017.12.002 30166925 PMC6111186

[3] Rasheed MK Alqasoumi A Hasan SS Babar ZUD. The community pharmacy practice change towards patient-centered care in Saudi Arabia: a qualitative perspective. J Pharm Policy Pract. (2020) 13:59. 10.1186/s40545-020-00267-7 32944258 PMC7488651

[4] Al-Arifi MN. Patients' perception, views and satisfaction with pharmacists' role as health care provider in community pharmacy setting at Riyadh, Saudi Arabia. Saudi Pharm J. (2012) 20:323–30. 10.1016/j.jsps.2012.05.007 23960807 PMC3745196

[5] El Hajj M Mansoor El Salem S. Public's attitudes towards community pharmacy in Qatar: a pilot study. Patient Prefer Adherence. (2011) 5:405–22. 10.2147/PPA.S22117 21949604 PMC3176180

[6] Almaghaslah D Alsayari A Asiri R Albugami N. Pharmacy workforce in Saudi Arabia: challenges and opportunities: a cross-sectional study. Int J Health Plann Manage. (2019) 34:e583–93. 10.1002/hpm.2674 30265404

[7] Almaghaslah D. Knowledge, attitude and practice of community pharmacists toward non-pharmaceutical products in Saudi Arabia. Front Public Health. (2022) 10:771308. 10.3389/fpubh.2022.771308 35570966 PMC9099022

[8] Almaghaslah D Alsayari A Almanasef M Asiri A. Cross-sectional study on pharmacy students' career choices in the light of Saudi Vision 2030: will community pharmacy continue to be the most promising, but least preferred, sector? Int J Environ Res Public Health. (2021) 18:4589. 10.3390/ijerph18094589 33926047 PMC8123572

[9] Almanasef M Almaghaslah D Kandasamy G Vasudevan R Batool S. Involvement of community pharmacists in public health services in Asir Region, Saudi Arabia: a cross-sectional study. Int J Clin Pract. (2021) 2021:e14940. 10.22541/au.162634177.72381621/v1 34606135

[10] Almaghaslah D Alsayari A Almaghaslah S Alsanna H. Patients' satisfaction with E-Prescribing (Wasfaty) in Saudi Arabia: a survey of country-level implementation. Healthcare. (2022) 10:806. 10.3390/healthcare10050806 35627943 PMC9141395

[11] Al-Tannir M Alharbi AI Alfawaz AS Zahran RI AlTannir M. Saudi adults satisfaction with community pharmacy services. Springerplus. (2016) 5:774. 10.1186/s40064-016-2442-8 27386260 PMC4912500

[12] El-Kholy AA Abdelaal K Alqhtani H Abdel-Wahab BA Abdel-Latif MMM. Publics' perceptions of community pharmacists and satisfaction with pharmacy services in Al-Madinah City, Saudi Arabia: a cross sectional study. Medicina. (2022) 58:432. 10.3390/medicina58030432 35334609 PMC8954639

[13] Madkhali OA Alzahrani F. Community pharmacists' perceptions of their role in provision of Anemia management in Jazan Region, Saudi Arabia, and the associated barriers. Healthcare. (2022) 10:1452. 10.3390/healthcare10081452 36011109 PMC9408312

[14] Almaghaslah D Alsayari A. Using a global systematic framework tool to identify pharmacy workforce development needs: a national case study on Saudi Arabia. RMHP. (2021) 14:3233–45. 10.2147/RMHP.S322577 34393530 PMC8354774

[15] Wazaify M. Societal perspectives on over-the-counter (OTC) medicines. Fam Pract. (2005) 22:170–6. 10.1093/fampra/cmh723 15710640

[16] Wazaify M Al-Bsoul-Younes A Abu-Gharbieh E Tahaineh L. Societal perspectives on the role of community pharmacists and over-the-counter drugs in Jordan. Pharm World Sci. (2008) 30:884–91. 10.1007/s11096-008-9244-1 18683077

[17] Cordina M McElnay JC Hughes CM. Societal perceptions of community pharmaceutical services in Malta. J Clin Pharm Ther. (1998) 23:115–26. 10.1046/j.1365-2710.1998.00142.x 9786097

[18] McElnay JC Nicholl AJ Grainger-Rousseau TJ. The role of the community pharmacist — a survey of public opinion in Northern Ireland. Int J Pharm Pract. (2011) 2:95–100. 10.1111/j.2042-7174.1993.tb00733.x30323391

[19] Aljadhey H Assiri GA Mahmoud MA Al-Aqeel S Murray M. Self-medication in Central Saudi Arabia: community pharmacy consumers' perspectives. SMJ. (2015) 36:328–34. 10.15537/smj.2015.3.10523 25737176 PMC4381018

[20] Beaver KM Al-Ghamdi MS Kobeisy AN. The effects of low self-control and delinquent peers on alcohol, tobacco, and drug use in a sample of Saudi Arabian Youth. Int J Offender Ther Comp Criminol. (2016) 60:1569–87. 10.1177/0306624X15583670 25906778

[21] Al-Haqwi AI. Perception among medical students in Riyadh, Saudi Arabia, regarding alcohol and substance abuse in the community: a cross-sectional survey. Subst Abuse Treat Prev Policy. (2010) 5:2. 10.1186/1747-597X-5-2 20092658 PMC2832638

[22] Anderson C Sharma R. Primary health care policy and vision for community pharmacy and pharmacists in England. Pharm Pract. (2020) 18:1870. 10.18549/PharmPract.2020.1.1870 32256901 PMC7092710

