# Supplementary material for: Attitudes of the general public toward community pharmacy services in Saudi Arabia: A cross-sectional study
Source: Front Public Health. 2023 Feb 24;11:1092215. doi: 10.3389/fpubh.2023.1092215 (PMC9998947; doi:10.3389/fpubh.2023.1092215)
Supplement: Supplementary file 1 [file Table_1.docx]

Appendix 1: Association between participants’ willingness to use community pharmacy services and demographic characteristics using Chi square test (n=449).

| Community pharmacy service | Gender | | Age group | | Educational level | |
| --- | --- | --- | --- | --- | --- | --- |
|  | Male | Female | 18-45 years | > 45 years | Diploma or less | Bachelor or postgraduate degree |
| **Selecting over-the-counter products**  Definitely/probably unwilling  Definitely/probably willing | 26 (19.7%)  106 (80.3%) | 44 (18.6%)  193 (81.4%) | 46 (16.1%)  239 (83.9%) | 24 (28.6%)  60 (71.4%) | 19 (18.3%)  85 (81.7%) | 51 (19.2%)  214 (80.8%) |
| *P*-value | 0.790 | | **0.011*** | | 0.830 | |
| **Management of minor ailments**  Definitely/probably unwilling  Definitely/probably willing | 27 (21.8%)  97 (78.2%) | 47 (19.3%)  197 (80.7%) | 46 (16.1%)  239 (83.9%) | 28 (33.7%)  55 (66.3) | 20 (19.2%)  84 (80.8%) | 54 (20.5%)  210 (79.5%) |
| *P*-value | 0.570 | | **0.000*** | | 0.792 | |
| **Selecting non-pharmaceuticals**  Definitely/probably unwilling  Definitely/probably willing | 33 (27%)  89 (73%) | 47 (20%)  188 (80%) | 50 (18.4%)  222 (81.6%) | 30 (35.3%)  55 (64.7%) | 24 (24.5%)  74 (75.5%) | 56 (21.6%)  203 (78.4%) |
| *P*-value | 0.130 | | **0.001*** | | 0.562 | |
| **Counselling on using medications**  Definitely/probably unwilling  Definitely/probably willing | 37 (28.5%)  93 (71.5%) | 54 (24.7%)  165 (75.3%) | 58 (22.2%)  203 (77.8%) | 33 (37.5%)  55 (62.5%) | 27 (26.2%)  76 (73.8%) | 64 (26%)  182 (74%) |
| *P*-value | 0.434 | | **0.005*** | | 0.969 | |
| **Medication therapy management**  Definitely/probably unwilling  Definitely/probably willing | 36 (32.7%)  74 (67.3%) | 68 (31.5%)  148 (68.5%) | 68 (27.8%)  177 (72.2%) | 36 (44.4%)  45 (55.6%) | 27 (29%)  66 (71%) | 77 (33%)  156 (67%) |
| *P*-value | 0.820 | | **0.005*** | | 0.482 | |
| **Dental health**  Definitely/probably unwilling  Definitely/probably willing | 39 (32.8%)  80 (67.2%) | 79 (35.7%)  142 (64.3%) | 87 (33.5%)  173 (66.5%) | 31 (38.8%)  49 (61.3%) | 39 (37.9%)  64 (62.1%) | 79 (33.3%)  158 (66.7%) |
| *P*-value | 0.583 | | 0.385 | | 0.420 | |
| **Screening for diseases**  Definitely/probably unwilling  Definitely/probably willing | 44 (37.9%)  72 (62.1%) | 86 (39.1%)  134 (60.9%) | 97 (37.6%)  161 (62.4%) | 33 (42.3%)  45 (57.7%) | 35 (35.7%)  63 (64.3%) | 95 (39.9%)  143 (60.1%) |
| *P*-value | 0.836 | | 0.454 | | 0.472 | |
| **Smoking cessation**  Definitely/probably unwilling  Definitely/probably willing | 43 (39.4%)  66 (60.6%) | 95 (43.6%)  123 (56.4%) | 103 (40.9%)  149 (59.1%) | 35 (46.7%)  40 (53.3%) | 42 (43.3%)  55 (56.7%) | 96 (41.7%)  134 (58.3%) |
| *P*-value | 0.476 | | 0.372 | | 0.794 | |
| **Disease counselling**  Definitely/probably unwilling  Definitely/probably willing | 53 (47.3%)  59 (52.7%) | 86 (42%)  119 (58%) | 104 (43.5%)  135 (56.5%) | 35 (44.9%)  43 (55.1%) | 37 (40.2%)  55 (59.8%) | 102 (45.3%)  123 (54.7%) |
| *P*-value | 0.357 | | 0.834 | | 0.405 | |
| **Healthy eating**  Definitely/probably unwilling  Definitely/probably willing | 53 (46.9%)  60 (53.1%) | 83 (41.5%)  117 (58.5%) | 100 (42%)  138 (58%) | 36 (48%)  39 (52%) | 40 (40.8%)  58 (59.2%) | 96 (44.7%)  119 (55.3%) |
| *P*-value | 0.354 | | 0.362 | | 0.526 | |
| **Physical activity promotion**  Definitely/probably unwilling  Definitely/probably willing | 51 (44%)  65 (56%) | 89 (44.3%)  112 (55.7%) | 104 (43.3%)  136 (56.7%) | 36 (46.8%)  41 (53.2%) | 45 (45%)  55 (55%) | 95 (43.8%)  122 (56.2%) |
| *P*-value | 0.957 | | 0.599 | | 0.839 | |
| **Vaccination & immunisation**  Definitely/probably unwilling  Definitely/probably willing | 61 (53.5%)  53 (46.5%) | 94 (44.3%)  118 (55.7%) | 104 (42.6%)  140 (57.4%) | 51 (62.2%)  31 (37.8%) | 44 (44%)  56 (56%) | 111 (49.1%)  115 (50.9%) |
| *P*-value | 0.114 | | **0.002*** | | 0.394 | |
| **Sexual health**  Definitely/probably unwilling  Definitely/probably willing | 58 (54.7%)  48 (45.3%) | 110 (53.4%)  95.1 (46.6%) | 118 (50.6%)  115 (49.4%) | 50 (63.3%)  29 (36.7%) | 48 (55.8%)  38 (44.2%) | 120 (53.1%)  106 (46.9%) |
| *P*-value | 0.825 | | 0.051 | | 0.667 | |
| **Weight management**  Definitely/probably unwilling  Definitely/probably willing | 72 (63.2%)  42 (36.8%) | 118 (57.6%)  87 (42.4%) | 142 (57%)  107 (43%) | 48 (41.7%)  22 (31.4%) | 51 (53.1%)  45 (46.9%) | 139 (62.3%)  84 (37.7%) |
| *P*-value | 0.329 | | 0.082 | | 0.124 | |
| **Counselling on alcohol dependence and drug misuse**  Definitely/probably unwilling  Definitely/probably willing | 96 (83.5%)  19 (16.5%) | 154 (74.4%)  53 (25.6%) | 175 (75.1%)  58 (24.9%) | 75 (84.3%)  14 (15.7%) | 72 (74.2%)  25 (25.8%) | 178 (79.1%)  47 (20.9%) |
| *P*-value | 0.061 | | 0.078 | | 0.334 | |

* Statistically significant association (*p*-value<0.05)

Appendix 2: Correlation between barriers and willingness to use community pharmacy services

|  | Lack of privacy in the pharmacy | Busyness of the pharmacist | Lack of awareness of the ability of the pharmacist | Rudeness of the pharmacist | Lack of trust in the pharmacist | Fear of asking the pharmacist |
| --- | --- | --- | --- | --- | --- | --- |
| **Selecting over-the-counter products**  Pearson Correlation  *P*-value | -0.026  0.576 | 0.000  0.996 | 0.065  0.172 | 0.015  0.753 | **0.179***  **0.000** | 0.022  0.649 |
| **Management of minor ailments**  Pearson Correlation  *P*-value | -0.055  0.246 | -0.014  0.760 | **0.094***  **0.046** | -0.017  0.717 | **0.231***  **0.000** | -0.020  0.679 |
| **Selecting non-pharmaceuticals**  Pearson Correlation  *P*-value | -0.011  0.820 | 0.029  0.543 | 0.066  0.166 | 0.054  0.257 | **0.241***  **0.000** | 0.019  0.685 |
| **Counselling on using medications**  Pearson Correlation  *P*-value | -0.009  0.857 | -0.076  0.110 | 0.055  0.243 | -0.054  0.253 | **0.216***  **0.000** | -0.041  0.382 |
| **Medication therapy management**  Pearson Correlation  *P*-value | -0.035  0.465 | -0.060  0.205 | 0.049  0.303 | -0.063  0.185 | **0.143***  **0.002** | -0.118  0.012 |
| **Dental health**  Pearson Correlation  *P*-value | -0.034  0.476 | -0.092  0.051 | 0.003  0.949 | -0.009  0.845 | **0.140***  **0.003** | -0.069  0.143 |
| **Screening for diseases**  Pearson Correlation  *P*-value | -0.019  0.689 | -0.080  0.090 | 0.051  0.285 | -0.022  0.638 | **0.134***  **0.004** | -0.080  0.089 |
| **Smoking cessation**  Pearson Correlation  *P*-value | -0.014  0.771 | -0.103  0.029 | -0.002  0.965 | -0.006  0.893 | **0.165***  **0.000** | -0.065  0.167 |
| **Disease counselling**  Pearson Correlation  *P*-value | -0.055  0.247 | -0.076  0.109 | 0.023  0.626 | 0.001  0.988 | **0.192***  **0.000** | -0.089  0.06 |
| **Healthy eating**  Pearson Correlation  *P*-value | -0.014  0.772 | -0.006  0.895 | -0.003  0.950 | -0.036  0.448 | **0.177***  **0.000** | -0.170  0.000 |
| **Physical activity promotion**  Pearson Correlation  *P*-value | -0.037  0.433 | -0.025  0.593 | 0.023  0.620 | -0.033  0.480 | **0.159***  **0.001** | -0.073  0.123 |
| **Vaccination & immunisation**  Pearson Correlation  *P*-value | 0.001  0.982 | -0.036  0.441 | 0.002  0.961 | -0.005  0.915 | **0.211***  **0.000** | -0.048  0.309 |
| **Sexual health**  Pearson Correlation  *P*-value | -0.014  0.766 | -0.065  0.172 | 0.033  0.483 | -0.05  0.292 | **0.109***  **0.021** | -0.112  0.018 |
| **Weight management**  Pearson Correlation  *P*-value | 0.037  0.430 | 0.016  0.741 | 0.036  0.441 | 0.028  0.560 | **0.164***  **0.000** | -0.060  0.203 |
| **Counselling on alcohol dependence and drug misuse**  Pearson Correlation  *P*-value | **0.096***  **0.043** | -0.025  0.596 | -0.020  0.669 | -0.011  0.810 | 0.012  0.806 | -0.056  0.235 |

* Statistically significant positive correlation (P-value<0.05)
